# p53 Abnormalities and Potential Therapeutic Targeting in Multiple Myeloma

**DOI:** 10.1155/2014/717919

**Published:** 2014-06-17

**Authors:** P. J. Teoh, W. J. Chng

**Affiliations:** ^1^Department of Medicine, Yong Loo Lin School of Medicine, National University of Singapore, Singapore 119077; ^2^Cancer Science Institute of Singapore, National University of Singapore, Singapore 117599

## Abstract

p53 abnormalities are regarded as an independent prognostic marker in multiple myeloma. Patients harbouring this genetic anomaly are commonly resistant to standard therapy. Thus, various p53 reactivating agents have been developed in order to restore its tumour suppressive abilities. Small molecular compounds, especially, have gained popularity in its efficacy against myeloma cells. For instance, promising preclinical results have steered both nutlin-3 and PRIMA-1 into phase I/II clinical trials. This review summarizes different modes of p53 inactivation in myeloma and highlights the current p53-based therapies that are being utilized in the clinic. Finally, we discuss the potential and promise that the novel small molecules possess for clinical application in improving the treatment outcome of myeloma.

## 1. Introduction

Located at the chromosome 17p13.1,* TP53* encodes for p53 tumor suppressor protein. Deemed the guardian of the genome, p53 safeguards the integrity of the genome and ensures that the tissue homeostasis is kept in check. Under normal physiological conditions, cellular p53 is expressed at low levels, thereby turning off the activity of p53 network. With stress induction, p53 is stabilized by means of posttranslational modifications, such as phosphorylation and acetylation [1, 2]. The subsequent accumulation of p53 in the nucleus ultimately results in the massive activation of the downstream signaling, whereby a range of diverse antiproliferation and proapoptotic genes are actively transcribed by the p53 transcription factor. These genes mediate tumor suppressive mechanisms such as the cell cycle arrest (p21, Gadd45, 14-3-3σ), senescence (p21), apoptosis (Bax, PUMA, Noxa), and inhibition of angiogenesis (TSP1, maspin) [1, 2].

Thirty years of intensive research on p53 have yielded significant understanding of its structure and basic functions. The high percentage of patients with p53 germline mutations succumbing to a wide range of cancer in Li-Fraumeni syndrome [3] is a testament of p53 being a critical tumor suppressor gene. Furthermore, the importance of p53 as a tumor suppressor is underscored by the fact that it is mutated in approximately 50% of human cancer [1, 2, 4, 5]. In contrast to all other types of human cancer, p53 abnormalities in hematological malignancies are uncommon events. This review summarizes the current knowledge about the p53 abnormalities in multiple myeloma (MM) and discusses the current and potential therapeutics targeting p53 abnormalities in this disease.

## 2. p53 Abnormalities in Multiple Myeloma

In MM, mutation of p53 gene is a rare occurrence at diagnosis; however, the incidence increases as the stage of disease advances, suggesting its essential role in disease progression [6–8]. Overall, p53 mutations were found to occur in about 3% of newly diagnosed patients [6–9]. The next generation sequencing methods that were recently employed into p53 mutational studies have also recapitulated low incidence rate of p53 mutations in MM [6, 7]. Nonetheless, it is often associated with poor prognosis and accounts for a significantly low survival rate [6, 8].

Deletion of chromosome 17p13 region, which contains the p53 gene locus, is a recurrent cytogenetic abnormality in MM and has been associated with less favorable outcome [6–11]. p53 deletion which was found to be predominantly monoallelic has a reported incidence rate ranging from 10% to 34% of the cases [6, 8, 12, 13]. In particular, this chromosomal abnormality was identified as one of the few factors that defined high risk and poor prognosis in MM [14]. In line with this, p53 deletion has been reported as an important factor associated with resistance to chemotherapy [15]. Furthermore, Chang et al. reported that myeloma patients with central nervous system involvement were found to have p53 deletion and this finding may suggest the association of this genetic abnormality with metastatic properties of myeloma cells [16]. Consistently, Elnenaei et al. and Billecke et al. also reported a higher percentage of patients with p53 deletions being in MM stage IIIb or having plasma cell leukemia, with advanced stage of organ infiltrations [15, 17]. Moreover, another recent study has also reported more rapid progression of MM to plasma cell leukemia in 17p13(del) cases as compared to patients without this abnormality [18]. Essentially, loss of p53 has also been reported to be important in the progression of MM which involved reprogramming of the hematopoietic progenitor cells to malignant plasma cells [19]. Therefore, these reports collectively highlighted the critical value of p53 deletion in the pathogenesis of MM.

Fifty percent of cancer harbours p53 mutations, while in the remaining 50%, the wild type p53 is deemed to lose its function via various mechanisms that affect the expression and activity of p53. The main inhibition mechanism of p53 has been described to be the amplification or overexpression of its negative regulator mouse double minute 2 homolog (MDM2). MDM2 is an E3 ubiquitin ligase which promotes proteasomal degradation of p53 as well as inhibiting the transactivation domain of the tumor suppressor protein [20–23]. Under normal physiological conditions, p53 is a labile protein with very short half-life ranging only from 5 to 30 minutes, owing to the incessant degradation by MDM2 [23]. Importantly, MDM2 itself is the product of a p53-inducible gene. Thus, the two molecules interact with each other through an autoregulatory negative feedback loop aimed at maintaining low cellular p53 levels in the absence of stress. Of importance, MDM2 has been found to be deregulated in various types of cancers, including MM [24–27]. Deregulation of MDM2 gene gives rise to the overexpression of its protein, thereby increasing the turnover rate of p53, keeping p53 level low at all times, and ultimately suppressing its tumor suppressive actions. In particular, overexpression of MDM2 was shown to be essential in promoting both the entry into cell cycle and tumor cell survival in myeloma cells [27].

MDM4, a homolog of MDM2, does not have E3 ubiquitin ligase activity but inactivates p53 by binding to and inhibiting the transactivation domain of p53. Due to its essential role in inactivating p53, MDM4 dysregulation in cancer has also been receiving important attention lately [24, 25]. In fact, MDM4 was said to enhance the E3 ligase activity of MDM2 and to increase p53 proteasomal degradation rate [21, 24]. This genetic abnormality is also relevant in the perspective of MM because amplification of chromosome 1q, a region at which the MDM4 gene resides, has been established as an independent and significant prognostic factor [28, 29]. Indeed, patients harbouring this abnormality are categorized in the subgroup of high risk MM [29].

On top of that, epigenetic regulation of* TP53* is also a subject of intense research of late. Deregulation of miRNAs in cancer is being rigorously explored and this has led to the hypothesis of the role of this group of noncoding genes in the pathogenesis of MM [30, 31]. miRNAs are a set of noncoding RNA sequence of 19 to 25 nucleotides that play a major role in regulating gene expression by degrading its target coding mRNA and by repressing protein translation through partial or complete base pairing to its complimentary sites on target mRNA [31]. miR-125b and miR-504 were described as bona fide negative regulators of p53 in human cell lines [32, 33]. Importantly in MM, studies on the miRNA regulation on p53 expression have identified that both miR-25 and miR-30d directly target the 3′-UTR of p53 mRNA and subsequently result in the decrease of p53 protein expression, depletion of the apoptosis response rate, and diminishment of cellular senescence [34]. Introduction of the inhibitors of miR-25 and miR-30d to a human myeloma cell line, NCI-H929, in turn increased the endogenous level of p53 protein, accompanied by the upregulation of proapoptotic gene PUMA and ultimately the increase of apoptosis [34].

In addition, epigenetic factors are also possible regulators of the expression and the activity of p53. For instance, the deregulation of p14ARF has been reported to be responsible in abolishing the integrity of the p53 pathway [35]. ARF has an essential role in downregulating the expression of MDM2, thereby reinstating the stability of p53 which then leads to the activation of its downstream pathway [2]. Hypermethylation of p14ARF has been described in various tumors [2, 36, 37] and more relevantly for this review, this epigenetic abnormality has been reported in MGUS and MM samples [38]. This finding reflects a situation where p14ARF hypermethylation occurs as an early event during the pathogenesis and development of MM.

Hypermethylation of the promoter region of* TP53* gene itself has also been demonstrated in human myeloma cell lines [39, 40]. Reversal of this epigenetic alteration by zebularine (DNA methyltransferase inhibitor) restored the expression of p53 in the cells followed by decreased cell viability and increased apoptosis [39, 40].

Collectively, these findings describe the diverse mechanisms of p53 inactivation in multiple myeloma.

## 3. p53 Reactivating Agents in Cancer and Myeloma

Due to the fact that p53 is the nexus of various tumor suppressive pathways, it is imperative to study the means of reactivating or restoring p53 functions in human cancer in order to revert or rescue cells from resistance towards standard chemotherapeutic treatments. In fact, many anticancer drugs induce apoptosis through multiple pathways that are at least in part dependent upon functional p53 activation. In the late 1980s and early 1990s, the introduction of wild type p53 gene into a variety of human tumor cells has shown induction of efficient growth inhibition and apoptosis [41]. In line with this, multiple efforts have been channeled into research for effective p53-based therapy. In fact, p53 gene therapy (Gendicine) has been approved as the standard treatment for a number of cancers in China [41].

In myeloma, a preclinical study demonstrated that adenovirus mediated delivery of wild type p53 could potently induce apoptosis in myeloma cells while sparing the normal hematopoietic cells and normal lymphocytes [42]. Furthermore, when p53 was ectopically reexpressed in human myeloma cell lines that are absent of p53 expression, a reduction in cell viability, with increased rate of apoptosis and cell cycle arrest, was observed [13]. These findings suggest that functional p53 pathways have a therapeutic effect on MM. Therefore, various drugs have been developed with this purpose of p53 pathway reactivation. The following section briefly describes the current p53-based antimyeloma therapies that are being administered in the clinic.

### 3.1. Current p53-Based Antimyeloma Therapy

#### 3.1.1. Chemotherapy

The use of conventional or high-dose chemotherapy has been a long-standing approach to treat MM patients. First line therapy for eligible patients remains autologous stem cell transplantation following high-dose chemotherapy. This is often preceded by induction therapy to decrease tumor load by utilizing a combination of treatments that often include chemotherapeutic agents such as alkylating compounds [43]. In relapsed or chemoresistant cases, combination chemotherapy is used as salvage therapy [44]. The most commonly prescribed chemotherapeutic drugs are as follows: melphalan, dexamethasone, prednisone, and etoposide [44]. Chemotherapy is useful in MM and other cancers due to its efficiency in killing malignant cells via a genotoxic mechanism. The majority of traditional chemotherapy agents target fast-growing tumor cells based on the notion that cancer cells are rapidly dividing and, therefore, are more sensitive to drugs that affect DNA replication. By mechanism, chemotherapy drugs potently induce DNA damage in cancer cells, thereby activating the p53 pathway which ultimately manifests as cell death [1, 2]. The importance of p53 in executing cytotoxicity is attested by the finding that p53 mutation and deletion conferred chemoresistance and significantly unfavorable outcome [6, 15]. In view of the mechanism of action of these genotoxic drugs targeting highly dividing cells, normal host cells that are rapidly growing are also damaged in the treatment process.

#### 3.1.2. Proteasome Inhibitors

Bortezomib was approved by the Food and Drug Administration (FDA) for MM treatment in 2008 [44]. Bortezomib belongs to a class of proteasome inhibitor. 26S proteasome is an enzyme complex located in the cytoplasm and nucleus of cells that degrades unneeded, damaged, or misfolded proteins that have been polyubiquitinated by E1, E2, and E3 ubiquitin ligases [45]. As mentioned in the earlier section, MDM2 is an E3 ubiquitin ligase that induces polyubiquitination of p53 protein and subsequently promotes its proteolytic degradation in the 26S proteasome complex, thereby keeping basal expression of p53 at bay under normal conditions [41, 46, 47]. In MM, overexpression of MDM2 will lead to a high production of polyubiquitinated p53 ready proteasomal degradation. In this instance, bortezomib was developed to rescue this mechanism by inhibiting and blocking the actions of proteasome, thus preventing p53 from being degraded [48–50]. Proteasome inhibition stabilizes p53 itself and its downstream targets such as p21 and Bax, resulting in halting of cell cycle progression and, ultimately, apoptosis [48–50]. Promising results arising from clinical trials have brought bortezomib into the clinic [51, 52]. The emergence of bortezomib represented a paradigm shift in the treatment of myeloma and has brought improved outcome and longer survival of MM patients [53]. Indeed, in MM patients, including both newly diagnosed and relapsed or refractory cases, bortezomib treatment demonstrated good efficacy with response rate (partial and complete response) ranging from 35% to as high as 80% [51–54]. However, like any other antimyeloma drug in the clinic, resistance towards bortezomib remains inevitable. Nearly a third of MM patients never respond to this drug treatment and those who responded initially developed resistance over time.

### 3.2. Potential Therapeutics Targeting p53

**Figure 1:**
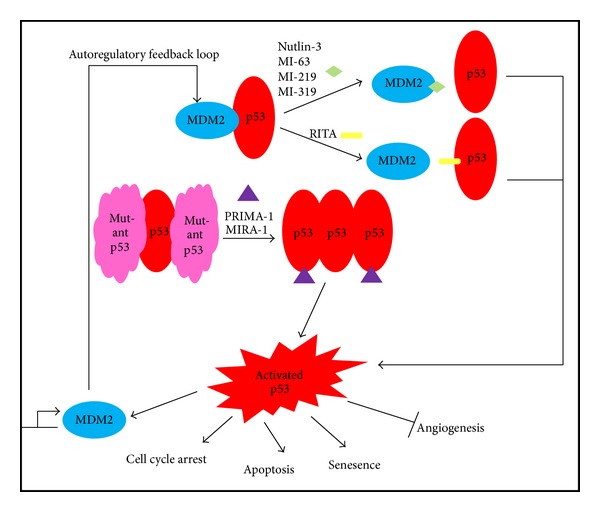
Schematic representation of mechanism of action of small molecules targeting p53 abnormalities. Simplified from [55].

Despite the introduction of bortezomib shifting the paradigm of MM treatment, the disease remains incurable and resistance towards this remarkable drug still arises. Given this situation, novel therapeutics targeting the critical p53 pathway is of utmost importance in order to reactivate the tumor suppressor network to execute apoptosis, in the hope of improving the treatment outcome in MM. In view of this, novel p53-reactivating agents have been developed and these agents are relevant to the nature and pathology of MM. The following describes these p53-reactivating drugs and the potential they hold for clinical applications.  Figure 1 depicts the mechanism of actions these drugs undertake in their course to reactivate the p53 pathway.

#### 3.2.1. Inhibitors of p53-MDM2 Interaction

Due to the fact that newly diagnosed cases of MM are often presented with wild type p53, therapeutic induction of p53 is an attractive potential treatment strategy for this disease. The conventional way of inactivating wild type p53 is through deregulation/overexpression of MDM2, which inhibits the transcriptional activity of p53 as well as increasing the rate of the p53 degradation. In view of this, the development of drugs to reactivate wild type p53 has focused on developing small molecule inhibitors to the MDM2-p53 complex.

*(i) Nutlin*. The first reported and the most well studied MDM2 inhibitor is the nutlins [29, 41]. Nutlins are a group of cis-imidazole analogs with high affinity for the p53-binding pocket on the amino terminal of MDM2 [56]. Nutlin was shown to resemble three important residues (Phe19, Trp23, and Leu26) on the transactivation domain of p53 that are critical for MDM2 binding [56]. In other words, nutlin competitively displaces p53 from the binding on MDM2 and effectively causes the stabilization of p53. Accumulation of p53 protein subsequently leads to its downstream pathway activation in cancer cells with wild type p53.

Because MDM2 inhibitors depend on p53 activation in cells expressing wild type p53, hematological malignancies that mostly retain wild type genotype of* TP53* are potential attractive targets for MDM2 inhibitor-based therapy. Nutlin-3 has been shown to be a potent inducer of apoptosis in cell lines deriving from hematological malignancies, including MM, ALL, AML, CLL, and Hodgkin's lymphoma [57, 58]. In MM, nutlin-3 demonstrated potent antimyeloma activity in MM cell lines, primary MM patient samples, as well as in MM cells grown in the bone marrow microenvironment [58, 59]. It was shown to reactivate the p53 pathway of the cells with wild type p53 by inducing the transcription of its downstream targets, p21 and MDM2, alongside the proapoptotic genes, PUMA, Bax, and Bak, which subsequently triggered cell death [58, 59]. This effect was observed specifically only in wild type but not in mutant p53 cells [58, 59]. The molecular mechanisms behind nutlin-induced apoptosis in MM were associated with both p53-transcription dependent and independent pathways [59]. Imperatively, nutlin was found to be lacking toxicity towards normal bone marrow hematopoietic cells [58]. In fact, this drug was demonstrated to have antigrowth instead of apoptosis-inducing effects on the normal hematopoietic stem cells [58]. This finding implicates the efficiency of nutlin acting as a nongenotoxic drug, killing myeloma cells while sparing normal host cells.

Furthermore, nutlin displayed wide synergy with various conventional chemotherapeutic drugs, namely, melphalan, etoposide, and velcade [58, 60, 61]. Taken together, these studies support the usage of nutlin as a novel treatment for MM either as a single agent or in combination with cytotoxic drugs. Nutlin could be utilized as a prechemotherapy agent in order to halt the growth of normal host cells, and subsequently standard and conventional chemotherapy drugs may be administered to induce cytotoxic death only to the cancerous cells. This strategy can be employed to minimize the chemotherapy-induced toxicity in normal dividing and growing cells, while concurrently tapering the high doses of cytotoxic chemotherapy.

As mentioned earlier, monoallelic deletion of p53 with the remaining allele being a wild type is a recurrent cytogenetic abnormality in MM patients and this group of patients often suffers from poor prognosis. It still remains inconclusive whether the single allele of wild type p53 in MM cells is actually still functional or is able to be reactivated by a nongenotoxic agent such as nutlin-3. Our lab has shown that, in WT/- cases with high p53 expression, nutlin-3 was able to induce a functional p53 pathway, but with a compromised activity compared to the WT/WT cells, whereas in WT/- cases with low or zero p53 expression, nutlin-3 showed no efficacy. The findings indicate a haploinsufficient activity of p53 in myeloma (P.J. Teoh et al. Leukemia in Press [62]).

The current evidence holds a lot of promise for nutlin-3 to be translated into the clinic as a treatment for MM with wild type p53. However, it must be kept in mind that sporadic mutations of p53 could arise from selective pressure in the cells upon prolonged nutlin treatment, rendering a state of resistance towards the drug [63]. Furthermore, overexpression of MDM4 (MDM2 homolog), another potent p53 negative regulator, could also bring about nutlin resistance. This follows the mechanism by which the freed p53 from MDM2 control could very possibly be in turn bound and inactivated by the high levels of MDM4. In fact, cells overexpressing MDM4 showed a decreased nutlin efficacy of inducing p53 activity, whereas silencing of the former enhanced the efficiency of nutlin in inducing apoptosis [64]. Even though MDM4 overexpression in MM is rare, its locus on chromosome 1q is frequently amplified [28]; thus it would be important not to rule out the possibility of nutlin resistance arising from this genetic deregulation. These findings also suggest the importance of designing small-molecule inhibitors of the MDM4-p53 interaction, or preferably, a dual inhibitor of both MDM2-p53/MDM4-p53 interactions to completely reactivate p53.

*(ii) RITA (Reactivation of p53 and Induction of Tumor Cell Apoptosis)*. RITA is a small molecule compound identified in a cell-based screen. It has a reversed mechanism from nutlin, whereby RITA binds to the amino terminal on p53 domain instead of on MDM2 protein. This binding causes conformational changes of p53 that reduces the p53-MDM2 interaction and hence decreases p53 ubiquitination which then leads to p53 accumulation, MDM2 downregulation, and p53-dependent apoptotic pathway induction [65]. Antimyeloma activity of RITA was first described in 2010 by Saha et al. by demonstrating that RITA also potently activates p53 pathway and showed efficient killing of myeloma cells with wild type p53, just like nutlin-3. Further validating the* in vitro* findings, mouse xenograft models of MM which were subjected to RITA treatment displayed tumor regression and lengthened survival [66].

The efficiency of RITA as an antimyeloma agent was further strengthened when RITA was found to be able to overcome resistance of MM cells towards MDM2 inhibitors such as nutlin-3 and MI-63 [67]. In this instance, RITA potently induced cell cycle arrest and apoptosis in resistant cells which were found to harbor p53 mutations after prolonged exposure to both nutlin-3 and MI-63 [67]. This piece of data suggests that RITA may very well possess a p53-independent role in exerting its antimyeloma activity. In line with this, another earlier study has reported a novel function of this compound in apoptotic signaling. Besides activating the p53 pathway, JNK signaling was also found to be induced upon RITA treatment, suggesting that this compound may function as a multitarget molecule [66]. Future investigations are needed to decipher this issue.

The clinical translation of RITA as an antimyeloma agent was further highlighted by its synergistic relationship with nutlin in inhibiting the growth and killing of MM cells [65].

*(iii) Other Small Molecules Inhibitors of p53-MDM2*. The importance of MDM2 inhibitors in hematological malignancies was emphasized when these compounds, MI-63, MI-219, and MI-319, showed preclinical efficacies [57]. Like nutlin, these MI compounds also bind to the p53 pocket on the surface of MDM2 only in cells with wild type p53 to reactivate the tumor suppressor pathway. MI-219 was shown to be effective in inducing apoptosis in p53 wild type cells of solid tumors originating from breast, colon, and prostate [41, 68]. The efficacy of MI-219 in hematological malignancy was evidenced when it was demonstrated to enhance the rate of MDM2 autoubiquitination, thereby increasing the degradation of this p53 negative regulator [69]. On the other hand, MI-319, which is a more potent derivative of MI-219, was found to be effective against another form of blood cancer, follicular lymphoma, with* in vitro* and* in vivo* evidence of reactivation of the p53 pathway [70]. These results suggest that the MI compounds could potentially be promising in myeloma although preclinical data is yet to be established.

#### 3.2.2. Reactivation of p53 Mutants

*(i) PRIMA-1 (p53 Reactivation and Induction of Massive Apoptosis)*. p53 mutations are often associated with resistance to chemotherapy treatment in cancer [6, 15]. These findings, together with the evidence that mutant p53 is often expressed at high levels, render mutant p53 as an important study target for cancer therapy. In view of this, p53 mutant reactivating agents have been developed. One such agent is the PRIMA-1. PRIMA-1 is a small molecule drug that reactivates mutant p53 by restoring its wild type conformation and transcriptional functions, consequently triggering massive apoptosis in tumor cells carrying mutant p53. Investigations into the molecular mechanism of the drug demonstrated that PRIMA-1 is converted into a by-product (methylene quinuclidinone) that forms adducts with thiols in mutant p53 [71]. This covalent modification of the mutant protein is sufficient to restore its binding ability to its transcriptional targets [71]. Since mutant p53 is often overexpressed in cancer cells, the restoration of wild type function in these high numbers of mutants ultimately triggers massive apoptosis, rendering this drug to be a highly effective anticancer strategy.

PRIMA-1 has been shown to have good efficacy against various types of solid cancer cells, namely, breast cancer [72], small cell lung carcinoma [73], and thyroid cancer [74]. This drug was able to reactivate the p53 pathway by inducing the transcription of various downstream targets (p21, MDM2, and Bax) and a consequent mutant-p53-dependent apoptosis [75]. It has also been shown that this drug has good antitumor effects at the* in vivo* level, whereby it potently inhibits the growth of tumor in human tumor xenograft model [73, 76]. PRIMA-1 was also shown to synergize with various chemotherapeutic agents to induce cancer cell death [77–79]. Due to its promising anticancer properties, PRIMA-1Met/APR246, a more potent derivative of the first generation drug, was developed and is currently in phase I/II clinical trials [80].

The potency of PRIMA-1 in hematological malignancies came to light when it was found to have an antileukemic effect in CLL and AML cells [78, 81]. Interestingly, it was found to be more cytotoxic to AML cell with hemizygous p53 deletion [81]. However, there is still very little information reporting antimyeloma activity of PRIMA-1 until recently it was shown to induce apoptosis in several human myeloma cell lines tested, irrespective of their p53 status [79]. Further investigation demonstrated that PRIMA toxicity was actually mediated by p73 (the p53 subfamily member) and Noxa [79]. This interesting finding denotes that the drug could be a versatile agent in treating MM patients with or without p53 abnormalities. Preliminary studies in our lab have revealed a p53-independent mechanism of PRIMA-1 in myeloma cell lines, consistent with the findings reported by Saha et al. [79]. Importantly, we also found that cell lines with no p53 expression were particularly more sensitive to the drug and when the response was further probed and elucidated, we found that the activation of the endoplasmic reticulum stress pathway seems to be the mechanism behind PRIMA-induced apoptosis. In fact Lambert et al. once reported that treatment of human sarcoma cell lines by PRIMA-1 induces multiple signaling pathways that eventually converge on a common apoptosis route, and ER stress was noted to be increased in response to the drug treatment as well [82]. This interesting finding calls for a more in-depth study to explore the drug efficacy, including its functional and biochemical effects in myeloma.

*(ii) MIRA-1*. MIRA-1, structurally distinct from PRIMA-1, is a maleimide compound that targets mutant p53 with higher potency than PRIMA-1 [83]. MIRA-1 was described to shift the equilibrium between the native and unfolded conformation of p53 towards the native conformation, leading to restoration of p53-mediated transactivation of target genes and induction of apoptosis in a mutant-p53-dependent manner [83]. First investigation of MIRA-1 in multiple myeloma was conducted by Saha et al. whereby, with resemblance to PRIMA-1 treatment, MIRA-1 showed antimyeloma activity independently of p53 status [57].

#### 3.2.3. Drug Combinations

Since drug resistance is ubiquitous in multiple myeloma, drug combination has been employed as a treatment regime to improve the treatment outcome. For instance, nutlin-3 has been shown to act in concert with various conventional chemotherapeutics to activate p53, in efforts to improve the treatment efficacy and reduce the collateral damage caused by chemotherapy. Nutlin-3 was reported to induce growth arrest in normal cells, and upon removal of the drug, cell cycle resumes [84]. As chemotherapy targets actively dividing cells, this mechanism of nutlin can be exploited to halt the growth of normal cells, preventing the toxicity caused by subsequent administration of chemotherapy.  Table 1 summarizes the combination therapies that have been reported to be effective in killing myeloma cells. The nongenotoxic small molecular agents, nutlin-3 and PRIMA-1, were shown to exert synergistic effects with a wide variety of chemotherapeutics. This indicates potential therapeutic efficacy utilizing these small molecules in the treatment of chemorefractory myeloma patients.

## 4. Conclusion and Future Directions

As p53 is the bridging point of apoptotic mechanisms, reactivation of the p53 pathway itself confers an excellent therapeutic approach in treating cancer. The current perspective points towards the importance of utilizing small molecular agents to reactivate the wild type p53 or to restore the transcriptional activities of the mutant p53. Even though each agent on its own was reported to show potent* in vitro* and* in vivo* antimyeloma effects, promising results arising from drug combinations suggest that combined use is a more attractive therapeutic option. The fact that p53-independent activities are involved in the drug mechanisms denotes that further investigations in this aspect are strongly called for so that we could fully utilize the versatility of the drug while maximizing its killing capacity. Of course, understanding the precise mechanism of action of these drugs would also aid in the future design of a novel and improved compound for the treatment of multiple myeloma.

## Figures and Tables

**Table 1:** Synergistic response of small molecules with various anticancer agents.

Drug	Synergistic drug	Target	Reference
Nutlin	Melphalan	WT p53, MDM2	[58]
Nutlin	Etoposide	WT p53, MDM2	[58]
Nutlin	Bortezomib	WT p53, MDM2	[60, 61]
Nutlin	Lexatumumab	WT p53, MDM2, DR5	[85]
Nutlin	RITA	WT p53, MDM2	[65]
RITA	MI-63	WT p53, mutant p53	[67]
PRIMA-1	Dexamethasone	p53-independent	[79]
PRIMA-1	Doxorubicin	p53-independent	[79]
